# An Audit of the Use of Barium Meal Examinations in General Practice

**Published:** 1992-03

**Authors:** J. E. Baldwin

**Affiliations:** Consultant Radiologist Yeovil District Hospital


### An Audit of the use of Barium Meal Examinations in General Practice

**J. E. Baldwin, MRCP, FRCR**
Consultant Radiologist
Yeovil District Hospital

An audit of the use of barium meal examinations in a six month period by two local practice surgeries (Table 1) was performed as a retrospective study based on the original requests, the findings of the examination and interviews with the general practitioners several months after the examinations were performed.

32 requests were made and 31 examinations undertaken. One patient was asymptomatic on attendance and the meal was not performed.

### RESULTS

The findings were classified into major abnormalities (i.e. those requiring specific treatment, such as ulceration) and minor abnormalities (i.e. those for which no specific treatment was required, such as minor oesophageal motility disorders) [Conry, McLean et al]. (Table 2). Major and minor abnormalities were found in 18 and 2 patients respectively and 11 patients had no abnormality.

The diagnosis made by the general practitioner was correct in a minority of cases (7 out of 19) in which a specific provisional diagnosis had been made. (Tables 3, 4 and 5).

The investigation was normal in 6 of 11 patients of those with epigastric pain but no specific provisional diagnosis. A variety of major abnormalities was demonstrated in the remainder. (Table 6).

The management of 20 of the patients was altered by the findings of the investigation. (Table 7).

The general practitioners found the results unhelpful in only two cases. In one, the report was difficult to interpret and in the other, a normal examination failed to reassure the patient.

### DISCUSSION

Barium meal examination continues to be useful in general practice, demonstrating a significant number of major abnormalities and affecting the management in the majority of cases. This study raised a number of questions.

1. It was difficult to know what provisional diagnosis had been made in some cases. (Table 6).
2. The classification of findings into major and minor abnormalities is debatable. For example, gastro-oesophageal reflux is classified as a major abnormality but can be an asymptomatic finding.
3. The reports were not always specific in distinguishing between acute and chronic and duodenual ulceration, when possible. Reports will be made as specific as possible in the future.
4. The retrospective analysis made it difficult to determine the true effect of the examination on the patient's management in some cases. A prospective study is necessary to answer some of these questions and this will be carried out as a future study.

### REFERENCE

CONRY B. G., McLEAN A. M., FARTHING M. J. G. Diagnostic and therapeutic efficacy of barium meal examination: A prospective evaluation in general practice. *British Medical Journal 1989;* **299:** 1443-5.

**Table 1**
**An Audit of the use of Barium Meal Examinations by two local Practice Surgeries**

The findings are based on a retrospective analysis of the results of all barium meal examinations (BAM) performed on patients referred from two local general practice surgeries from January to June 1990 inclusive.

**Patients details**

| | | |
|---|---|---|
| Number of patients ... ... ... ... ... | **Surgery A** | **18** |
| ,, ,, ... ... ... ... ... | **Surgery B** | **14** |
| Ages of patients (years) ... ... ... | **50** | **16** |
| ,, ,, ... ... ... | **50** | **16** |
| Sex of patients ... ... ... ... ... | **Male** | **13** |
| ,, ,, ... ... ... ... ... | **Female** | **19** |

**Table 2**
**BAM findings in 32 patients**

| | Total | Normal | Major Abnormality | Minor Abnormality |
|---|---|---|---|---|
| Patients aged 50 | 16 | 4 | 10 | 2 |
| Patients aged 50 | 16* | 7 | 8 | 0 |
| Patients taking H2 receptor antagonists | 12 | 4 | 8 | 0 |

*included one on whom a BAM was not performed as the patient was asymptomatic at the time of the appointment.

**Major abnormalities** were assessed as those probably requiring specific medical treatment (for example ulcer, reflux, oesophagitis and erosions) or a surgical or endoscopic intervention (for example stricture, polyps) and minor abnormalities as incidental findings (for example uncomplicated small hiatus hernia and minor oesophageal motility disorder).

**Table 3**
**Indications for BAM**

**Provisional diagnosis based on clinical symptoms and signs**

| | |
|---|---|
| Gastro-oesophageal reflux (GOR)/oesophagitis | 3 |
| Oesophageal obstruction | 1 |
| Hiatus hernia (HH) | 1 |
| Ulcer — specified | 9 |
| Carcinoma | 3 |
| Mass | 2 |
| "Epigastric pain" | 11 |
| Reassurance | 4 |
| Unclear/unstated | 2 |

**Table 4:**
**Findings of Barium Meal Examinations**

| | |
|---|---|
| GOR/Oesophagitis ... ... ... ... ... ... | 6 |
| Oesophageal obstruction ... ... ... ... ... | 0 |
| HH ... ... ... ... ... ... ... ... ... | 2 |
| Impaired oesophageal motility ... ... ... ... | 1 |
| Ulcer ... ... ... ... ... ... ... ... | 7 |
| Duodenitis ... ... ... ... ... ... ... | 1 |
| Carcinoma ... ... ... ... ... ... ... | 0 |
| Mass ... ... ... ... ... ... ... ... | 0 |
| Polyps ... ... ... ... ... ... ... ... | 2 |
| Normal ... ... ... ... ... ... ... ... | 12 |
| Not Examined ... ... ... ... ... ... ... | 1 |
| TOTAL | 32 |

**Table 5**
**The Accuracy of the Clinical Diagnosis**

| Provisional diagnosis made by GP | Total | Accuracy Correct | Incorrect | BAM findings in incorrect diagnosis |
|---|---|---|---|---|
| GOR/Oesophagitis | 3 | 1 | 2 | 1 normal<br>1 gastric polyp |
| Oesophageal Obstruction | 1 | 0 | 1 | 1 GOR |
| HH | 1 | 1 | 0 | |
| Ulcer | 9 | 5 | 4 | 4 normal |
| Carcinoma | 3 | 0 | 3 | 2 normal<br>1 HH |
| Mass | 2 | 0 | 2 | 1 normal<br>1 HH |

**Table 6:**
**The findings of BAM with Epigastric Pain and no specific provisional diagnosis**

| | |
|---|---|
| Normal | 6 |
| GOR | 2 |
| HH | 1 |
| Gastric Polyps | 1 |
| Duodenitis | 1 |
| TOTAL | 11 |

**Table 7:**
**Effect of Barium Meal findings on Patients' Treatment**

| | |
|---|---|
| Changed ... ... ... ... ... ... ... | 14 |
| Unchanged ... ... ... ... ... ... ... ... | 10 |
| Treatment stopped ... ... ... ... ... ... ... | 2 |
| Gastroscopy ... ... ... ... ... ... ... ... | 4 |
| Unknown ... ... ... ... ... ... ... ... | 1 |
| Not Examined ... ... ... ... ... ... ... | 1 |

